# Systematic review and meta‐analysis of intravenous iron therapy for adults with non‐anaemic iron deficiency: An abridged Cochrane review

**DOI:** 10.1002/jcsm.13114

**Published:** 2022-11-02

**Authors:** Cory Dugan, Katerina Cabolis, Lachlan F. Miles, Toby Richards

**Affiliations:** ^1^ Division of Surgery, Faculty of Health and Medical Science The University of Western Australia Perth Australia; ^2^ Department of Neuroinflammation, UCL Queen Square Institute of Neurology University College London London UK; ^3^ Department of Critical Care, Faculty of Medicine, Dentistry and Health Sciences The University of Melbourne Melbourne Australia

**Keywords:** anaemia physical function, fatigue, iron deficiency, sarcopenia

## Abstract

Iron is an essential nutrient for oxygen supply and aerobic metabolism. Iron deficiency impacts cellular respiration and mitochondrial energy metabolism, which can lead to reduced skeletal muscle function and muscle mass, causing sarcopenia. Intravenous iron offers the ability to rapidly correct iron deficiency, but the functional impact on patient mental and physical health is unclear. We assessed the effects of intravenous iron therapy on physical function and quality of life in the treatment of adults with non‐anaemic iron deficiency. An update and reanalysis of a previously published Cochrane systematic review was performed to assess randomized controlled trials that compared any intravenous iron preparation with placebo in adults. The primary functional outcome measure was physical performance as defined by the trial authors. Secondary outcome measures included fatigue and quality‐of‐life scores, and adverse effects at the end of follow‐up. Biochemical efficacy was assessed by change in serum ferritin and haemoglobin concentration levels. Twenty‐one randomized controlled trials, comprising 3514 participants, were included. Intravenous iron compared with placebo resulted in significantly increased physical function measured by mean peak oxygen consumption (mean difference [MD] 1.77 mL/kg/min, 95% confidence interval [CI] 0.57 to 2.97). An overall improvement in fatigue was seen (standardized MD 0.30, 95% CI −0.52 to −0.09) but no overall difference in quality of life (MD 0.15, 95% CI −0.01 to 0.31). Biochemically, intravenous iron resulted in improved serum ferritin (MD 245.52 μg/L, 95% CI 152.1 to 338.9) and haemoglobin levels (MD 4.65 g/L, 95% CI 2.53 to 6.78). There was a higher risk of developing mild adverse events in the intravenous iron group compared with the placebo group (risk ratio 1.77, 95% CI 1.10 to 2.83); however, no differences were seen in serious adverse events (risk difference 0, 95% CI −0.01 to 0.01). The quality of evidence was rated ‘low’ and ‘very low’ for all outcome variables, except for fatigue, mainly due to most studies being judged as having a high risk of bias. In non‐anaemic iron‐deficient adults, the use of intravenous iron compared with placebo improved physical function and reduced fatigue scores. However, we remain uncertain about the efficacy in this population due to low‐quality evidence, and there is a need for further studies to address potential impact on overall quality of life.

## Introduction

Iron deficiency is the most common nutritional deficiency and cause of anaemia worldwide.^1^ The World Health Organization (WHO) regards iron deficiency anaemia as a key contributor to disability, with negative associations on exercise tolerance and mental well‐being.^2^, ^3^, ^4^, ^5^, ^6^ However, the impact of iron deficiency *without* anaemia on physical function is less well defined.

**Figure 1:**
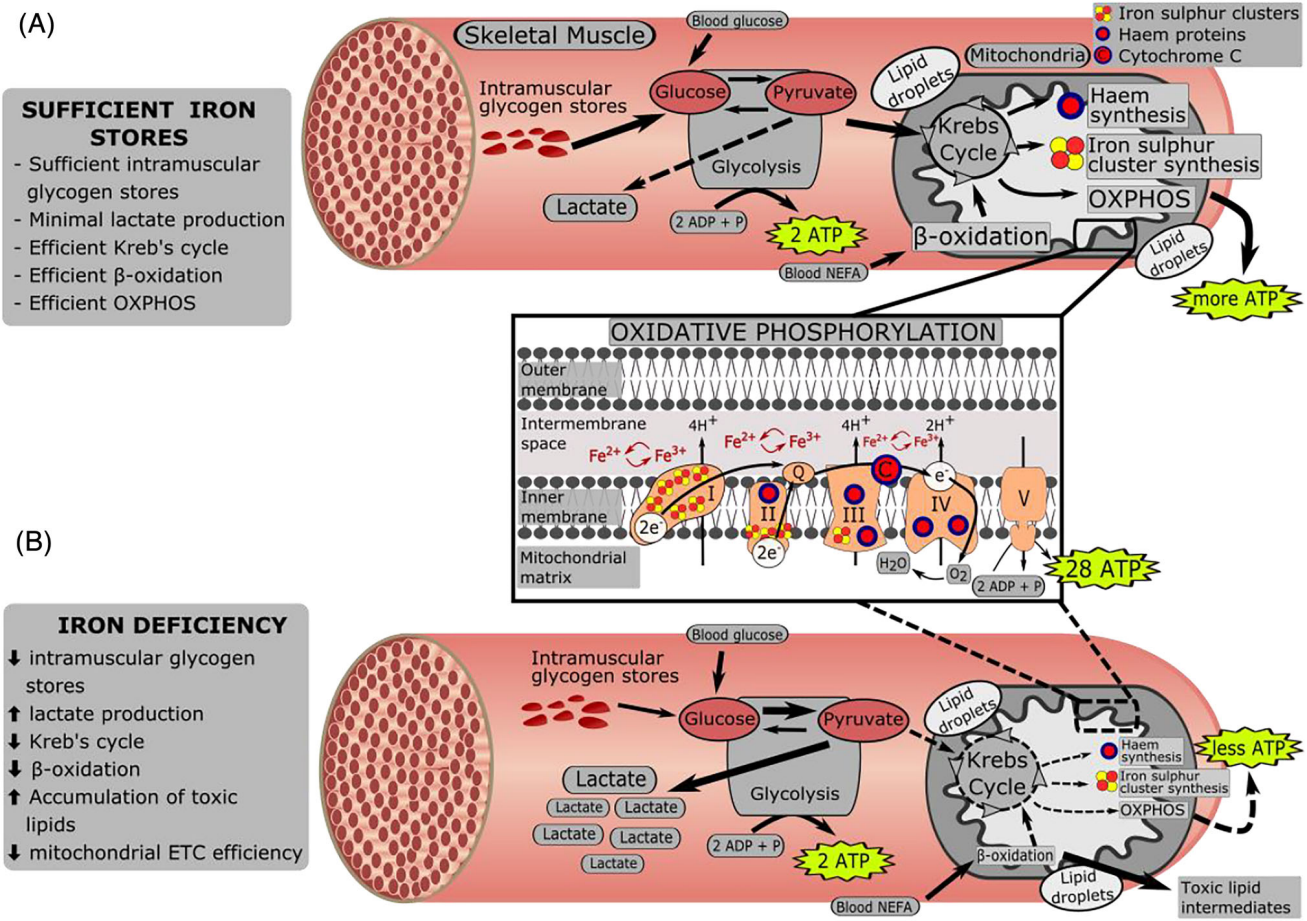
Diagram depicting the energetic pathway of skeletal muscle tissue (A) in a state of sufficient iron stores and (B) in a state of iron deficiency; there is a decrease in glycogen stores, an increase in lactate production, a decline in the Krebs cycle and oxidative phosphorylation and thereby lower levels of ATP overall. Oxidative phosphorylation panel highlights the role of iron in the electron transport chain. ADP, adenosine diphosphate; ATP, adenosine triphosphate; C, cytochrome c; e, electrons; ETC, electron transport chain; Fe^2+^, ferrous iron; Fe^3+^, ferric iron; H, hydrogen; H_2_O, water; I–IV, mitochondrial complexes I–IV; NEFA, non‐esterified fatty acids; O_2_, oxygen; OXPHOS, oxidative phosphorylation; P, phosphate; Q, coenzyme Q

Iron is a fundamental micronutrient^7^, ^8^ essential for the transport and storage of oxygen, as well as mitochondrial energy production,^9^, ^10^, ^11^, ^12^, ^13^, ^14^ which generates adenosine triphosphate (ATP) through aerobic respiration (oxidative phosphorylation) in the electron transport chain. This is particularly relevant for skeletal muscle, which contains up to 10–15% of total body iron^15^ and requires adequate levels of iron to support healthy mass and function. Iron deficiency can reduce the cellular ability for aerobic respiration, leading to compromised ATP production, which, in turn, is associated with a decrease in aerobic capacity and muscle function (*Figure* *1*).^11^, ^12^ These aetiological processes have implicated iron deficiency as a contributing factor for the development, and recovery, from sarcopenia.^16^, ^17^

Intravenous iron has become an established treatment option to rapidly replenish iron stores and effective treat iron deficiency anaemia.^18^, ^19^, ^20^ However, the evidence supporting the use of intravenous iron in non‐anaemic iron‐deficient adults, specifically the impact on physical function and performance, is equivocal,^21^ with clinical trials reporting both increases^22^ and no change^19^, ^23^ to exercise capacity. In a recent Cochrane review, low‐quality evidence in the included studies reporting maximum oxygen consumption (VO_2_ max) and quality‐of‐life measures meant that appropriate analysis could not be conducted accurately. This was in the most part due to heterogeneity in research protocols that included different participant populations, with variation in the definition of iron deficiency, and used different doses of iron or different modalities of inconsistent administration. When focusing solely on women populations of reproductive age, previous meta‐analysis has demonstrated improvements in maximal and submaximal physical function following iron supplementation.^24^ However, this review did not investigate parenteral iron therapies. Hence, the effect of intravenous iron therapy on physical function in non‐anaemic iron‐deficient individuals remains unresolved.^21^ Despite this, the use of intravenous iron continues to increase in developed countries,^25^ with multiple ‘best practice’, consensus statements and guidelines advocating for its use.^25^, ^26^, ^27^ The aforementioned lack of empirical evidence is particularly detrimental to women's health, given that women are more likely to have iron deficiency compared with men when adopting equal reference ranges.^28^ Consequently, the current standard of care for women with iron deficiency has been called into question, with evidence suggesting the need to single women out and investigate further, rather than accept the dogma of normality.^29^

To better understand the evidence driving recommendations and clinician behaviours, we assessed the effects of intravenous iron therapy in adults with non‐anaemic iron deficiency by renewing and reanalysing the results of a previous Cochrane review^21^ with primary focus on the effect of intravenous iron on physical function and quality of life.

## Methods

The Cochrane methodology was applied to this review.^21^ *Table* *1* presents the inclusion and exclusion criteria against which studies were screened.

**Table 1:** Inclusion and exclusion criteria

Inclusion criteria	
Study type	Randomized control trials (irrespective of blinding, language, study setting or sample size).
Publication	Trials were included irrespective of publication status and date of publication.
Participants	Any non‐pregnant and non‐lactating adults with functional or absolute iron deficiency, without anaemia. No anaemia defined as Hb > 130 g/L for men and Hb > 120 g/L for women.
Intervention and comparisons	Any formulation of intravenous iron versus placebo.
Outcome measures	Primary: Any measure of physical/muscle function, defined by the trial authors (e.g., VO_2_ max, 6MWT and muscle function). Secondary: Haemoglobin concentration, measured at the end of follow‐up; serum ferritin, measured at the end of follow‐up; fatigue scores, measured using quantitative measurement scales taken at the end of follow‐up; overall quality of life, measured using a quantitative measurement scale taken at the end of follow‐up; risk of serious adverse events at end of follow‐up, defined as any events that would increase mortality, were life threatening, required inpatient hospitalization or resulted in persistent or significant disability, or any medical events that might jeopardize the participants at the required intervention to prevent them within 30 days of cessation of treatment; and risk of mild adverse events at the end of follow‐up, defined as any events that did not meet the definition of a serious adverse event but that required treatment or resulted in patient discomfort; hypophosphataemia of any severity was included in this category.

Exclusion criteria	
Study type	Quasi‐randomized trials, cross‐over trials, non‐RCT designs.
Types of participants	Pregnant and puerperal women, paediatric populations, participants who were tested with erythropoietin or other erythropoiesis‐stimulating agents (ESA) alone or in combination with iron.
Types of intervention	Oral iron preparations.

Abbreviations: 6MWT, 6‐min walk test; Hb, haemoglobin; RCT, randomized controlled trial.

The Cochrane Injuries Group's Information Specialist searched the following databases on 18 October 2019 in accordance with the Cochrane Handbook for Systematic Reviews of Interventions^30^: Cochrane Central Register of Controlled Trials (which contains the Cochrane Injuries Trials Register; CENTRAL; 2019, Issue 10) in the Cochrane Library; MEDLINE Ovid (1946 to October 2019); Embase Ovid (1947 to October 2019); Web of Science: Science Citation Index Expanded (SCI‐EXPANDED; 1970 to October 2019); Web of Science: Conference Proceedings Citation Index‐Science (CPCI‐S; 1990 to October 2019); Clinicaltrials.gov (www.clinicaltrials.gov); and WHO International Clinical Trials Registry Platform (ICTRP; www.who.int/ictrp). A second search was performed on 15 July 2021, which aimed to find new studies that had been published since the previous search. This search used an identical strategy. The reference lists of all included studies and previously published reviews were searched for additional studies. Full details of the search strategy are available (Appendix S1).

### Study selection

All randomized controlled trials (RCTs) designs examining intravenous iron preparations versus placebo were considered for inclusion in this review. Specifically, RCTs were included irrespective of blinding, language of publication, publication status, date of publication, study setting or sample size. Quasi‐randomized trials, cross‐over trials and other non‐RCT designs were not included. Quasi‐randomized trials were defined as any controlled trial where the method of allocation was not truly random (i.e., allocation based on medical record number, date of birth and day of week). Cluster‐randomized trials were considered for inclusion if the method of randomization was truly random (i.e., random number sequence and coin flip). Finally, cross‐over trials were excluded as it is considered as an inappropriate design to assess this intervention.

All adults (18 years and above) with functional or absolute non‐anaemic iron deficiency were included. Non‐anaemic iron deficiency was defined as having a haemoglobin concentration > 130 g/L for men and >120 g/L for non‐pregnant women. Studies that did not differentiate haemoglobin concentration levels between men and women and set a non‐anaemic definition of >120 g/L for both sexes were also included. In order to capture the broadest possible population, a series of RCTs from the existing literature was reviewed to define iron deficiency and chose the least restrictive definition.^31^ Iron deficiency was defined as follows:

1. absolute: ferritin < 100 μg/L; and
2. functional: ferritin more than 100 μg/L and transferrin saturation (TSAT) < 20%.

### Assessment of the risk of bias

Included studies were assessed for risk of bias according to the criteria outlined in tab. 8.5.d in the Cochrane Handbook for Systematic Reviews of Interventions.^30^ The domains used to assess the risk of bias were selection bias (random sequence generation and allocation concealment), blinding bias (blinding of participants and personnel and blinding of outcome assessment), attrition bias (amount, nature and handling of incomplete outcome data), reporting bias (selective reporting of outcome data) and other bias (bias not covered elsewhere such as source of funding bias). Two review authors (CD and KC) identified studies for inclusion independently of each other. Disagreements were resolved through discussion or, if required, through involvement of a third review author (LFM).

### Statistical analysis

Meta‐analyses were performed using the software package Review Manager Version 5.3^32^ and in accordance with the recommendations of the Cochrane handbook.^30^ All effect estimates were calculated using a random effects model. Different treatment effects were used depending on the type of data. For continuous outcomes, using the inverse variance method, the mean difference (MD) or standardized mean difference (SMD) with 95% confidence intervals (CIs) were calculated where appropriate.

All SMD calculations were re‐expressed in units of the commonest scale in accordance with guidance from the Cochrane handbook. As several trials used different scales to assess physical function (6‐min walk test, fibromyalgia impact questionnaire [FIQR] walk score, short form [SF12] physical score and peak oxygen consumption) at different time points, and due to the lack of response from relevant authors for data, an analysis was conducted on all physical function outcomes irrespective of units or scale. This was achieved by calculating the SMD of each variable with respect to the change from baseline, which was re‐expressed back into peak oxygen consumption (mL/kg/min) using a typical SD from the included studies,^33^ in accordance with the Cochrane handbook. Several trials also used a variety of scales to measure fatigue scores (Piper fatigue score, visual numeric scale [VNS], numeric rating scale [NRS], multidimensional fatigue symptom inventory [MFSI], brief fatigue inventory [BFI] and fatigue severity scale [FSS]). Consequently, SMD was calculated and re‐expressed back into the Piper Fatigue Scale using a typical SD from the included studies.^18^ For similar reasons, SMD was calculated for the quality‐of‐life measurements (EQ‐5D, Kidney Disease Quality of Life [KDQoL] instrument, Minnesota Living with Heart Failure Questionnaire [MLFHQ], SF12 mental score, International Restless Legs Scale [IRLS], Kansas City Cardiomyopathy Questionnaire [KCCQ] and chronic obstructive pulmonary disease [COPD]), which was then re‐expressed back into the EQ‐5D, using a typical SD from the included studies.^34^

For dichotomous outcomes, the Mantel–Haenszel technique was used. The risk ratio (RR) and 95% CIs were calculated for binary variables, except for serious adverse events, which involved the calculation of risk difference (RD) along with the 95% CIs.

Due to the lack of common protocols used in the research studies, a certain amount of heterogeneity was expected in the analysis. This was related to a number of factors, including marked differences in study population (ranging from athletes^19^ to individuals with heart failure^35^) and the differing preparations and dosages of iron between studies. Consequently, the chi‐squared (*χ* ^2^) test was employed to explore heterogeneity of included studies with a significant alpha level of 0.05 determined a priori. We also measured heterogeneity using the *I* ^2^ statistic.^36^ Further, sensitivity analysis was conducted to assess the impact of varying definitions of iron deficiency on all outcomes. Specifically, studies that included patients with TSAT < 20% regardless of ferritin levels were excluded in this analysis, due to the possibility of varying iron deficiency aetiology (functional vs. absolute).

### Summary of findings and assessment of the certainty of the evidence

The results of this review for all comparisons are displayed in a ‘Summary of findings’ table (*Table* *2*). The primary outcome was MD in physical function (peak oxygen consumption: VO_2_ peak), taken at the end of follow‐up. The following secondary outcomes were also assessed:

1. SMD from baseline in physical function, as defined by the trial authors;
2. MD in concentration of ferritin (μg/L), taken at the end of follow‐up;
3. MD in concentration of haemoglobin (g/L), taken at the end of follow‐up;
4. SMD in fatigue scores, taken at the end of follow‐up;
5. SMD in quality‐of‐life scores, taken at the end of follow‐up; and
6. risk of mild adverse events.

The ‘Summary of findings’ table was prepared using GRADEpro GDT software (GRADEpro GDT). In accordance with the GRADE approach, we undertook an assessment of the quality of evidence for each outcome. We examined the risk of bias within studies, as well as the directness of evidence, heterogeneity, precision of effect estimates and risk of publication bias. The quality of evidence was graded as ‘high’, ‘moderate’, ‘low’ or ‘very low’.^37^

**Table 2:** Summary of findings table

Intravenous iron compared with placebo for non‐anaemic iron‐deficient adults					
**Population**: Non‐anaemic, iron‐deficient adults					
**Setting**: All healthcare setting (acute, subacute and community care)					
**Intervention**: Intravenous iron					
**Comparison**: Placebo					

Outcomes	Anticipated absolute effects (95% CI)		Relative effect (95% CI)	Number of participants (studies)	Certainty of the evidence (GRADE)
	Risk with placebo	Risk with intravenous iron			
Physical function (peak oxygen consumption: VO_2_ peak) (mean)	27.26 mL/kg/min	MD 1.77 mL/kg/min higher (0.57 higher to 2.97 higher)	—	194 (4 RCTs)	⨁◯◯◯ Very low^a^ ^,^ ^b^
Physical function (change from baseline) (SMD)	—	SMD 0.68 higher (0.01 higher to 1.35 higher)	—	639 (7 RCTs)	⨁◯◯◯ Very low^a^ ^,^ ^c^
Ferritin concentration (mean)	82.95 μg/L	MD 245.52 μg/L higher (152.11 higher to 338.94 higher)	—	1242 (12 RCTs)	⨁◯◯◯ Very low^a^ ^,^ ^d^
Haemoglobin concentration (mean)	131.76 g/L	MD 4.65 g/L higher (2.53 higher to 6.78 higher)	—	1675 (15 RCTs)	⨁⨁◯◯ Low^a^ ^,^ ^e^
Fatigue scores taken at the end of follow‐up	—	SMD 0.3 lower (0.52 lower to 0.09 lower)	—	814 (5 RCTs)	⨁⨁⨁◯ Moderate^a^
Quality‐of‐life scores taken at the end of follow‐up	—	SMD 0.15 higher (0.01 lower to 0.31 higher)	—	1030 (8 RCTs)	⨁⨁◯◯ Low^a^ ^,^ ^f^
Mild adverse events (risk ratio)	248 per 1000	439 per 1000 (273 to 701)	RR 1.77 (1.10 to 2.83)	1412 (11 RCTs)	⨁⨁◯◯ Low^a^ ^,^ ^e^

*Note*: The risk in the intervention group (and its 95% confidence interval) is based on the assumed risk in the comparison group and the relative effect of the intervention (and its 95% CI). GRADE Working Group grades of evidence: High certainty, we are very confident that the true effect lies close to that of the estimate of the effect; moderate certainty, we are moderately confident in the effect estimate—the true effect is likely to be close to the estimate of the effect, but there is a possibility that it is substantially different; low certainty, our confidence in the effect estimate is limited—the true effect may be substantially different from the estimate of the effect; very low certainty, we have very little confidence in the effect estimate—the true effect is likely to be substantially different from the estimate of effect. Abbreviations: CI, confidence interval; MD, mean difference; RCTs, randomized controlled trials; RR, risk ratio; SMD, standardized mean difference.

^a^ Downgraded one level for risk of bias: Several of the included studies were either unclear or at a high risk of bias.

^b^ Downgraded two levels for imprecision: The point prevalence estimates in each of the included studies are highly imprecise, as reflected by the large confidence interval of the total result.

^c^ Downgraded two levels for inconsistency: There was substantial statistical heterogeneity in the pooled results and multiple points of methodological heterogeneity.

^d^ Downgraded two levels for inconsistency: There was substantial statistical heterogeneity in the pooled results and multiple points of methodological heterogeneity, with dose of iron administered and time to the end of follow‐up.

^e^ Downgraded one level for inconsistency: There was substantial statistical heterogeneity in the pooled results.

^f^ Downgraded one level for imprecision: The total result confidence interval is wide and crosses the line of no effect.

## Results

### Study selection

**Figure 2:**
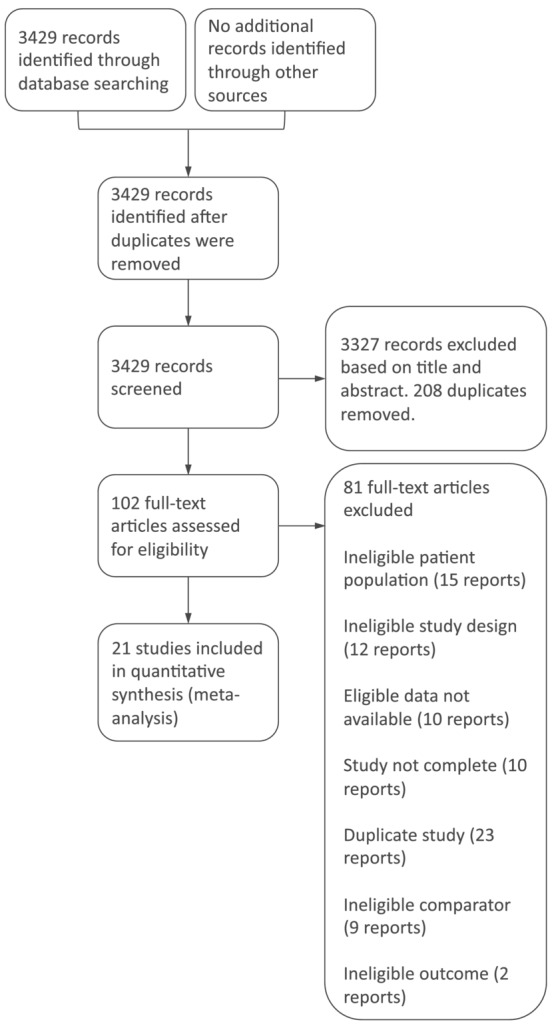
Flow diagram of studies included in the systematic review and meta‐analysis

The conducted search yielded 3429 references. Following de‐duplication and primary screening, 102 articles were selected for full‐text screening, and 21 studies were included in the qualitative and quantitative analyses.^18^, ^19^, ^23^, ^33^, ^34^, ^35^, ^38^, ^39^, ^40^, ^41^, ^42^, ^43^, ^44^, ^45^, ^46^, ^47^, ^48^, ^49^, ^50^, ^51^, ^52^ Exclusions are detailed in *Figure* *2*.

### Study characteristics

The included studies reported results for 3514 participants. Of these studies, seven were in people with heart failure,^33^, ^35^, ^41^, ^44^, ^47^, ^51^, ^52^ two in elite athletes,^19^, ^23^ two in otherwise well, pre‐menopausal women,^18^, ^46^ two in people with restless legs syndrome^42^, ^50^ and two in blood donors,^34^, ^43^ and the remaining six were a variety of specific cohorts (following cardiac surgery, fibromyalgia, kidney disease, complex vascular heart surgery, COPD and cardiac transplant recipients).^38^, ^39^, ^40^, ^45^, ^48^, ^49^ The commonest placebo comparator used was sodium chloride (0.9%), and two studies were open‐label interventions.^47^, ^51^

**Figure 3:**
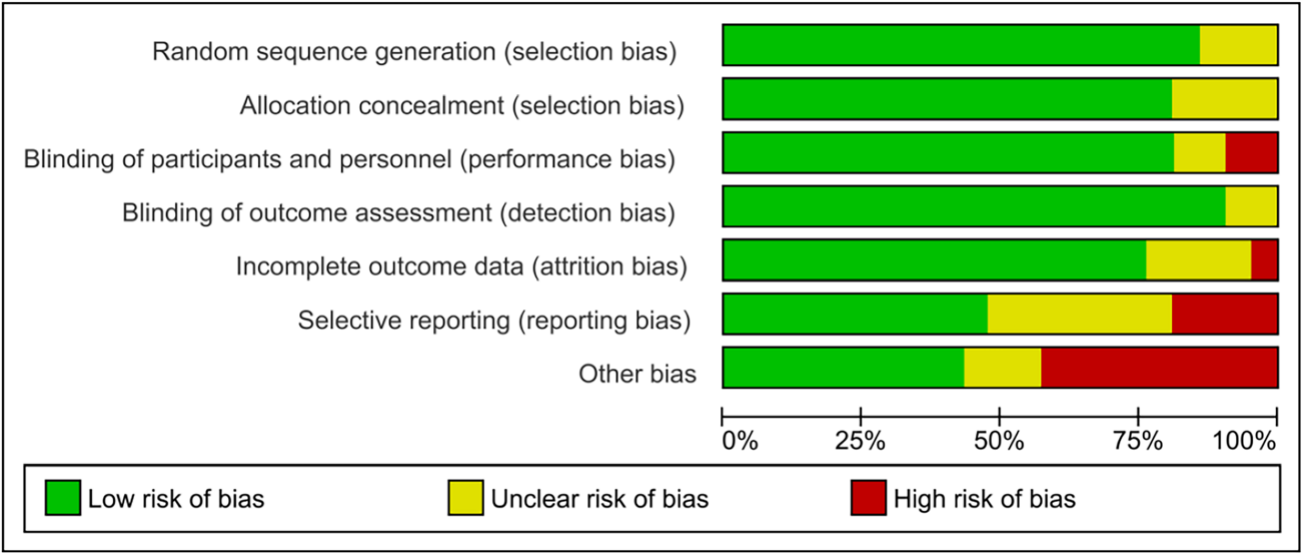
‘Risk of bias’ graph: Review authors' judgements about each ‘risk of bias’ item presented as percentages across all included studies

**Figure 4:**
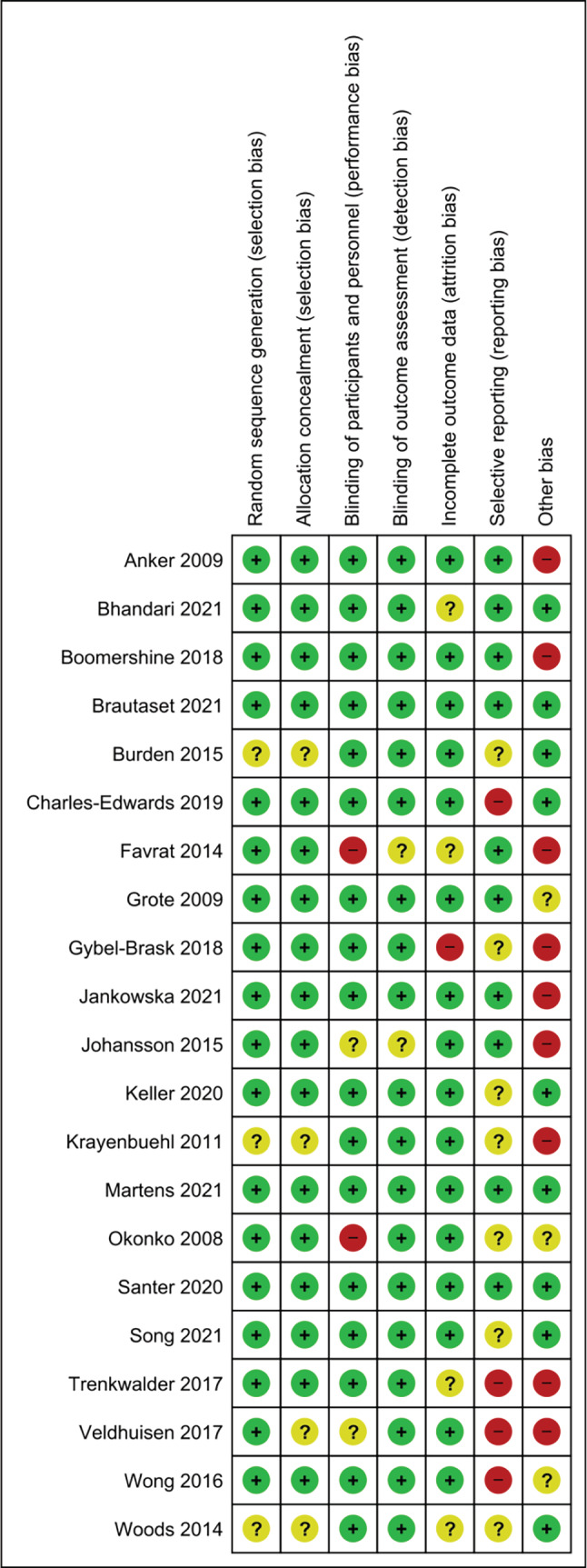
‘Risk of bias’ summary: Review authors' judgements about each ‘risk of bias’ item for each included study

All studies were available through database searches as full manuscripts, with the exception of Wong et al., which was a conference extract.^52^ The risk of bias graph is illustrated in *Figure* *3*, and a summary of the risk of bias analysis is presented in *Figure* *4*. Further description of study characteristics can be found in *Table* *3*.

**Table 3:** Summary of literature included in the meta‐analysis

Author(s)	Participant cohort (number, sex)	Population	Definition of iron deficiency	Iron preparation	Dosing regimens	Frequency	Total dose	Follow‐up
Anker 2009	*n* = 459 (*n* = 244 F)	Heart failure	Ferritin < 100 or 100–200 μg/L where TSAT < 20%	FCM	200 mg	Weekly until replete, then monthly	—	24 weeks
Bhandari 2021	*n* = 54 (*n* = 27 F)	Kidney disease	Ferritin < 100 μg/L and/or TSAT < 20%	Ferric derisomaltose/iron isomaltoside	1000 mg	Single dose	1000 mg	3 months
Boomershine 2018	*n* = 81 (*n* = 80 F)	Fibromyalgia	Ferritin < 50 μg/L and/or TSAT < 20%	FCM	15 mg/kg (≤750 mg)	Two doses over 5 days	≤1500 mg	42 days
Brautaset Englund 2021	*n* = 102 (*n* = 37 F)	Cardiac transplant recipients	Ferritin < 100 or 100–300 μg/L where TSAT < 20%	Ferric derisomaltose	20 mg/kg	Single dose	—	6 months
Burden 2015	*n* = 15 (*n* = 9 F)	Elite athletes	Ferritin < 30 μg/L for women and <40 μg/L for men	FCM	500 mg	Single dose	500 mg	4 weeks
Charles‐Edwards 2019	*n* = 40 (*n* = 11 F)	Heart failure	Ferritin < 100 or 100–200 μg/L where TSAT < 20%	Iron isomaltoside	608 ± 204 mg	Single dose	608 ± 204 mg	2 weeks
Favrat 2014	*n* = 290 F	Pre‐menopausal women	Ferritin < 15 or <50 μg/L where TSAT < 20%	FCM	1000 mg	Single dose	1000 mg	8 weeks
Grote 2009	*n* = 60 (*n* = 53 F)	Restless legs syndrome	Ferritin < 45 μg/L	FCM	200 mg	Five doses over 2 weeks	1000 mg	11 weeks
Gybel‐Brask 2018	*n* = 107 F	Blood donors	Ferritin < 60 μg/L	Iron isomaltoside	1000 mg	Single dose	1000 mg	10 weeks
Jankowska 2021	*n* = 1058 (*n* = 477 F)	Heart failure	Ferritin < 100 or 100–300 μg/L where TSAT < 20%	FCM	1000 mg	Two doses over 6 weeks	1000 mg	42 days
Johansson 2015	*n* = 60 (*n* = 8 F)	Post‐cardiac surgery	Ferritin < 100 or 100–200 μg/L where TSAT < 20%	Iron isomaltoside	1000 mg	Single dose	1000 mg	4 weeks
Keller 2020	*n* = 405 (*n* = 187 F)	Blood donors	Ferritin < 50 μg/L	FCM	800 mg	Single dose	800 mg	6–8 weeks
Krayenbuehl 2011	*n* = 90 F	Pre‐menopausal women	Ferritin < 50 μg/L	Iron sucrose	200 mg	Four doses over 2 weeks	800 mg	12 weeks
Martens 2021	*n* = 75 (*n* = 24 F)	Heart failure	Ferritin < 100 or 100–300 μg/L where TSAT < 20%	FCM	500–2000 mg	Single dose or two doses over 2 weeks	—	3 months
Okonko 2008	*n* = 35 (*n* = 10 F)	Heart failure	Ferritin < 100 or 100–200 μg/L where TSAT < 20%	Iron sucrose	200 mg	Weekly until replete, then monthly	—	18 weeks
Santer 2020	*n* = 48 (*n* = 14 F)	Chronic obstructive pulmonary disease	Ferritin < 100 or 100–300 μg/L where TSAT < 20%	FCM	1000 mg	Single dose	1000 mg	8 weeks
Song 2021	*n* = 204 (*n* = 123 F)	Complex valvular heart surgery	Ferritin < 100 μg/L and/or TSAT < 20%	Iron isomaltoside	≤1000 mg	Two doses given 3 days before and after surgery	≤2000 mg	3 weeks
Trenkwalder 2017	*n* = 110 (*n* = 90 F)	Restless legs syndrome	Ferritin < 75 or 75–300 μg/L where TSAT < 20%	FCM	1000 mg	Single dose	1000 mg	4 weeks
Van Veldhuisen 2017	*n* = 172 (*n* = 43 F)	Heart failure	Ferritin < 100 or 100–200 μg/L where TSAT < 20%	FCM	500–1000 mg	Six‐weekly until Week 12	1000–2500 mg	24 weeks
Wong 2016	*n* = 35	Heart failure	Ferritin < 100 or 100–200 μg/L where TSAT < 20%	FCM	1000 mg	Single dose	1000 mg	4 weeks
Woods 2014	*n* = 14 (*n* = 8 F)	Elite athletes	Ferritin 30–100 μg/L	FCM	100 mg	Three fortnightly doses over 4 weeks	300 mg	6 weeks

Abbreviations: F, females; FCM, ferric carboxymaltose; TSAT, transferrin saturation.

### Intervention

Studies used a variety of different intravenous iron group treatment regimens for the administration of the study drug. Twelve studies used a single‐dose administration,^18^, ^23^, ^33^, ^34^, ^38^, ^40^, ^41^, ^43^, ^45^, ^48^, ^50^, ^52^ whereas nine used repeat dosing at various points throughout the study.^19^, ^35^, ^39^, ^42^, ^44^, ^46^, ^47^, ^49^, ^51^ Ferric carboxymaltose was used in thirteen studies,^18^, ^19^, ^23^, ^33^, ^34^, ^35^, ^39^, ^42^, ^44^, ^48^, ^50^, ^51^, ^52^ iron isomaltoside (ferric derisomaltose) was used in five studies,^40^, ^41^, ^43^, ^45^, ^49^ both preparations (ferric carboxymaltose and ferric derisomaltose) were used together in one study,^38^ and iron sucrose was used in two studies.^46^, ^47^ The total dose of intravenous iron administered, where calculation was possible, ranged from 300^19^ up to 2500 mg.^51^

### Physical function

#### Peak oxygen consumption

**Figure 5:**
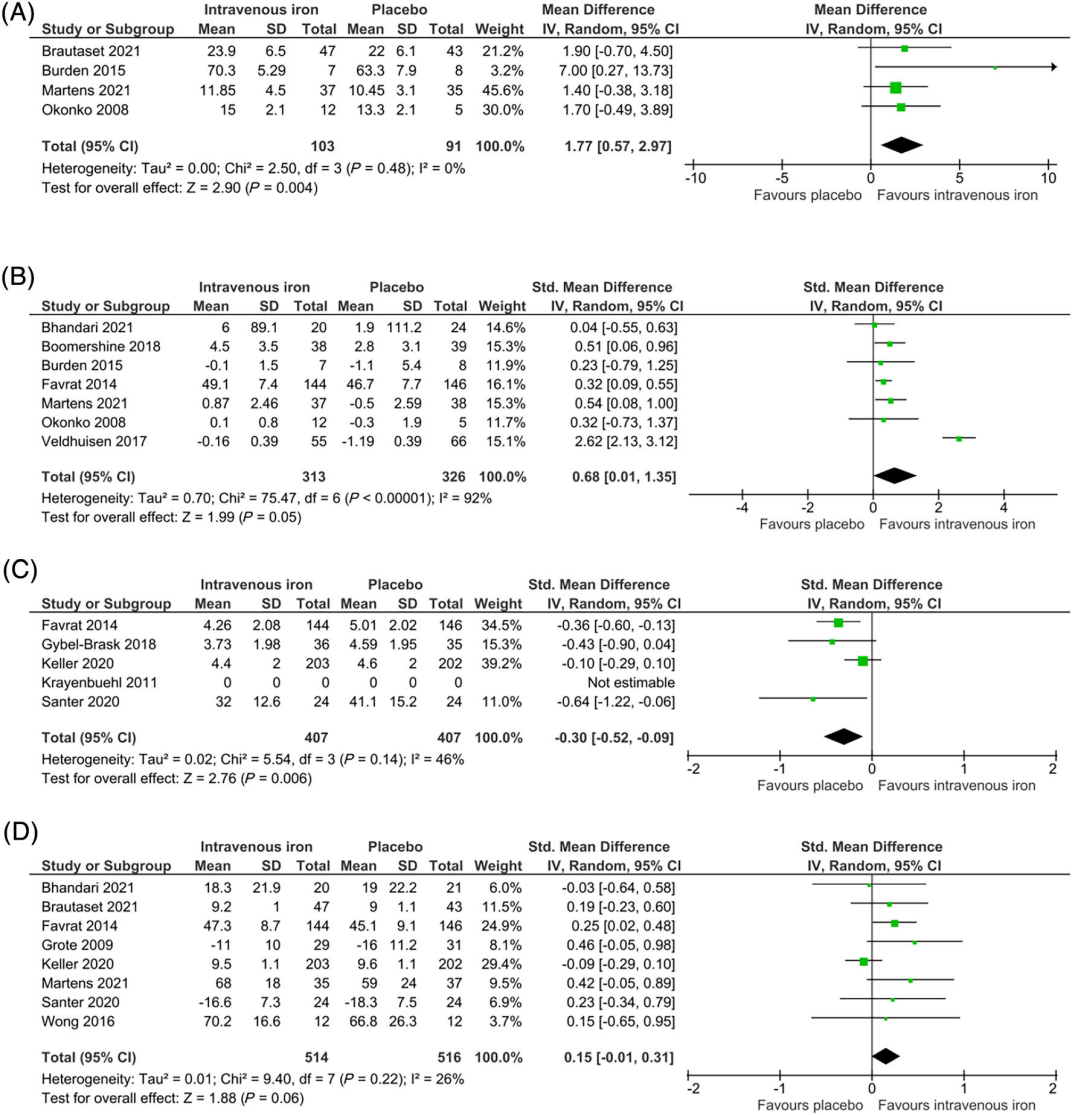
Intravenous iron versus placebo forest plots. Squares indicate study‐specific mean difference (MD) or standardized mean difference (SMD) estimates; horizontal lines indicate the 95% confidence interval (CI); diamonds indicate the pooled MD or pooled SMD with their 95% CIs. (A) Peak oxygen consumption (MD). (B) Physical function as defined by trial authors (SMD). (C) Fatigue at the end of follow‐up (SMD). (D) Quality of life at the end of follow‐up (SMD)

Four studies had endpoints that reported peak oxygen consumption measured at the end of follow‐up (*Figure* *5* *A*). Peak oxygen consumption taken at the end of follow‐up in the intervention group was on average 1.77 mL/kg/min higher than that of placebo (95% CI 0.57 to 2.97; *I* ^2^ = 0%; 4 studies, 194 participants; *P* = 0.004), but with ‘very low’ quality of evidence.

#### Physical function as defined by trial authors (standardized mean difference)

Seven studies, with a variety of assessments, reported physical function change relative to baseline scores (*Figure* *5* *B*). Meta‐analysis suggested that the mean physical function score was 0.68 SMD units higher in the intravenous iron group compared with that of placebo (95% CI 0.01 to 1.35; 7 studies, 639 participants; *P* = 0.05). Modelling the effect seen from the included studies (SD),^33^ this effect was re‐expressed into mL/kg/min (peak oxygen consumption value). Overall, the effect of intravenous iron was an increase in peak oxygen consumption value by an MD of 1.76 mL/kg/min higher compared with placebo (95% CI 0.03 to 3.50). Considerable heterogeneity was present in this analysis (*I* ^2^ = 92%; *χ* ^2^ = 75.47, *P* < 0.00001), with ‘very low’ quality of evidence.

### Fatigue at the end of follow‐up

Five trials reported findings for fatigue at the end of follow‐up, using a variety of scales (*Figure* *5* *C*). Intravenous iron was associated with reduced fatigue. Meta‐analysis suggested that the levels of fatigue taken at the end of follow‐up were 0.30 SMD units lower in the intervention group (95% CI −0.52 to −0.09; *I* ^2^ = 46%; 5 studies, 814 participants; *P* = 0.006). On average, the intravenous iron group scored 0.61 lower in the Piper Fatigue Scale (95% CI −1.05 to −0.18), implying lower fatigue compared with placebo, with ‘moderate’ quality of evidence.

### Quality of life at the end of follow‐up

Eight trials included findings for quality of life using a variety of scales (*Figure* *5* *D*). Overall, the effect of intravenous iron compared with placebo was not significant in terms of quality of life at the end of follow‐up (SMD 0.15; 95% CI −0.01 to 0.31; *I* ^2^ = 26%; 8 studies, 1030 participants; *P* = 0.06). When re‐expressing the results to generic quality‐of‐life scales (EQ‐5D), a similar outcome was seen with improved scores in the intravenous iron group compared with placebo; however, this was not significant (MD 0.17; 95% CI −0.01 to 0.34 higher; *P* = 0.06), with ‘low’ overall quality of evidence.

### Haemoglobin concentration

**Figure 6:**
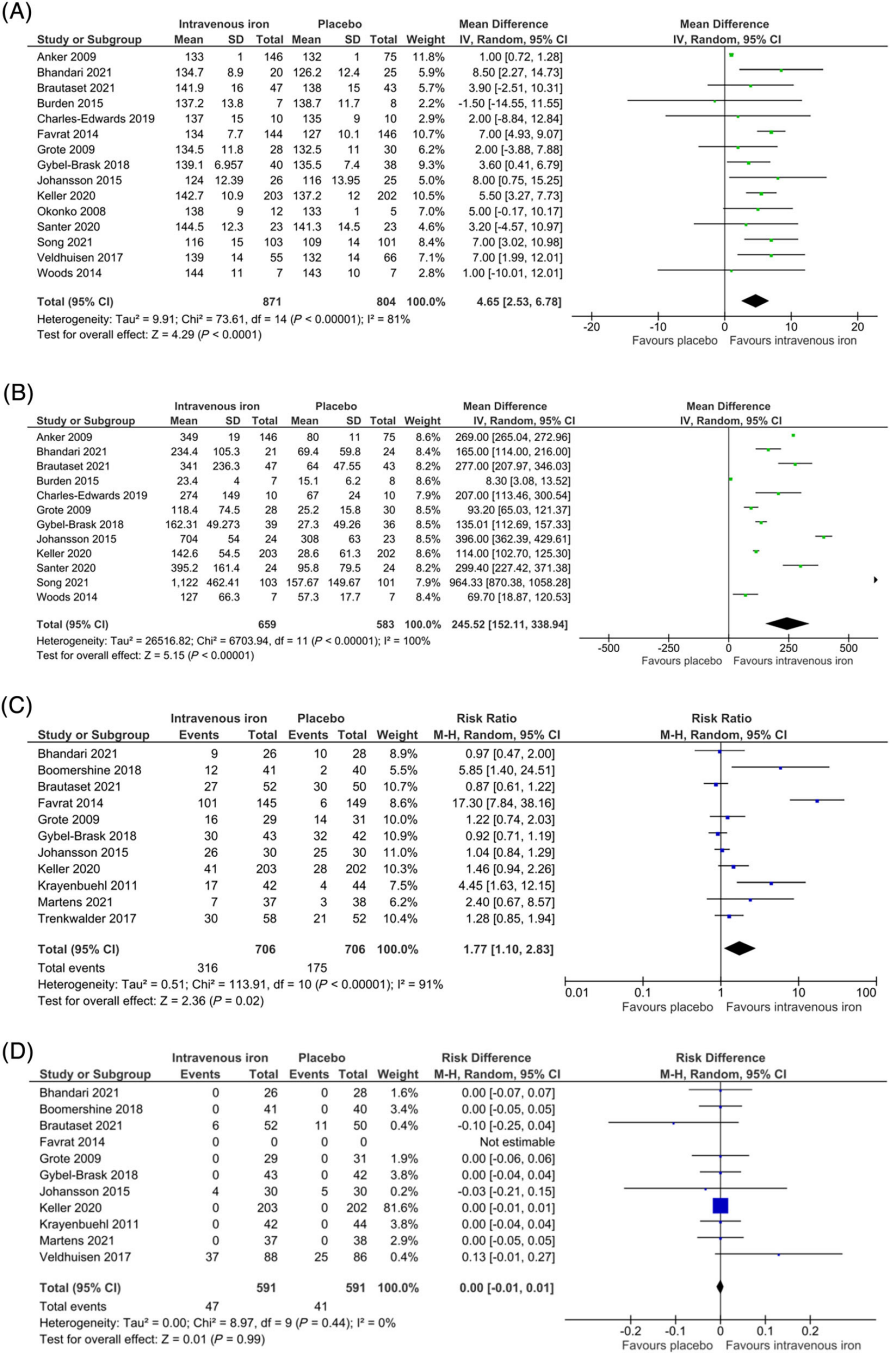
Intravenous iron versus placebo forest plots. Squares indicate study‐specific mean difference (MD) or standardized mean difference (SMD) estimates or pooled risk ratios (RRs) or pooled risk differences (RDs) estimates; horizontal lines indicate the 95% confidence interval (CI); diamonds indicate the pooled MD or pooled SMD with their 95% CIs. (A) Haemoglobin concentration at the end of follow‐up (MD). (B) Ferritin concentration at the end of follow‐up (MD). (C) Mild adverse events at the end of follow‐up. (D) Serious adverse events at the end of follow‐up (RDs)

Fifteen studies reported haemoglobin concentration at the end of follow‐up (*Figure* *6* *A*). Intravenous iron resulted in a higher haemoglobin concentration relative to the placebo taken at the end of follow‐up (MD 4.65 g/L; 95% CI 2.53 to 6.78; 15 studies, 1675 participants; *P* < 0.0001). Considerable heterogeneity was present in this analysis (*I* ^2^ = 81%; *χ* ^2^ = 73.61, *P* < 0.00001), with ‘low’ quality of evidence.

### Ferritin concentration at the end of follow‐up

Twelve studies reported ferritin concentration at the end of follow‐up (*Figure* *6* *B*). The MD in ferritin concentration taken at the end of follow‐up was 245.52 μg/L higher in the intervention group relative to the placebo group (95% CI 152.11 to 338.94; 12 studies, 1242 participants; *P* < 0.00001). Considerable heterogeneity was present in this analysis (*I* ^2^ = 100%; *χ* ^2^ = 6703.94, *P* < 0.00001), with ‘very low’ quality of evidence.

### Mild adverse events

Eleven trials included data corresponding to mild adverse events (*Figure* *6* *C*). Intravenous iron resulted in a higher rate of mild adverse events relative to placebo (RR 1.77; 95% CI 1.10 to 2.83; 11 studies, 1412 participants; *P* = 0.02). Considerable heterogeneity was present in this analysis (*I* ^2^ = 91%; *χ* ^2^ = 113.91, *P* < 0.00001), with ‘low’ quality of evidence.

### Serious adverse events

Eleven studies included data for serious adverse events (*Figure* *6* *D*). No differences were seen between intravenous iron relative to placebo (RD 0; 95% CI −0.01 to 0.01; *I* ^2^ = 0%, 11 studies, 1182 participants; *P* = 0.99), with ‘low’ quality of evidence.

### Sensitivity analysis

All relevant analyses were repeated with the exclusion of trials that included patients with TSAT < 20%, regardless of ferritin levels. No significant alterations were demonstrated in the results of all analyses.

## Discussion

In this updated Cochrane systematic review, of 21 studies, with 3514 participants with non‐anaemic iron deficiency, the use of intravenous iron compared with placebo resulted in improved physical function by recorded or modelled effect on peak oxygen consumption and reduced fatigue levels compared with placebo. However, no overall difference was seen in reported overall quality of life. The efficacy of intravenous iron compared with placebo in non‐anaemic iron deficiency was associated with increased serum ferritin concentration and haemoglobin concentration.

The present review builds upon the previously conducted Cochrane review^21^ with the addition of new evidence of larger studies that allowed for improved quality evidence. Specifically, this enabled the determination of improved fatigue scores in response to intravenous iron treatment. To the best of our knowledge, this updated review is the first meta‐analysis to confirm evidence of efficacy of intravenous iron for improved fatigue scores in non‐anaemic iron‐deficient adults. This is an important finding due to the significant increase in the use of intravenous iron globally in the last decade.^25^, ^26^ Further, this finding is of particular significance to women's health, where the current standard of care has been questioned and, subsequently, termed ‘misogynistic’.^29^ The improved quality evidence from the present review should better inform ‘best practice’, consensus statements and guidelines concerning the use of intravenous iron.

Impact of intravenous iron on muscle function and performance has demonstrated a mix of results, with studies reporting both increases^22^, ^24^, ^53^ and no change in exercise capacity in response to iron therapy.^54^, ^55^, ^56^ The divergence in findings have been explained, at least in part, by a recent prospective case control study, which demonstrated that although non‐anaemic iron‐deficient individuals treated with intravenous iron showed no differences in both aerobic respiration and VO_2_ max, intravenous iron increased lactate threshold during exercise, implying an increased ability to generate work aerobically with increasing exercise intensities in the absence of fatigue.^57^ These findings are in keeping with previous physiological experiments in animals with clinical implications.^13^ Further research investigating changes in lactate concentration in iron‐deficient individuals treated with intravenous iron is needed to confirm this.

We acknowledge some limitations. Despite biological plausibility, and seemingly ‘positive’ results in some of the included trials, we were unable to reach robust conclusions as to the role of intravenous iron therapy in physical function in non‐anaemic iron‐deficient adults. This was primarily due to the often variable statistical and methodological heterogeneity. Several differences between studies regarding population demographics, as well as intravenous iron regime, dose and frequency, were evident, all of which likely contributed to the heterogeneity of results. Also, the definition of iron deficiency was highly variable across studies, including serum ferritin < 100, <50, <30 or <15 μg/L, with or without a TSAT < 20%, possibly further confounding the conclusions due to potentially differing iron deficiency aetiology (i.e., functional vs. absolute). Further to this, several studies were deemed to be high risk of bias. As a result, the overall quality of evidence for many of the outcomes was graded as either ‘low’ or ‘very low’, apart from fatigue scores, which were graded as ‘moderate’. This highlights the need to standardize trial endpoints with harmonized trial protocols as seen in the clinical literature.^58^ Finally, in addition to the aforementioned limitations, significant difficulties were encountered when extracting data. Studies frequently reported outcomes in differing ways (i.e., absolute values at follow‐up vs. change from baseline), which consequently saw them excluded from the analysis despite considerable efforts being made to contact the authors and resolve these difficulties.

In conclusion, the appropriateness of intravenous iron therapy for the treatment of non‐anaemic iron deficiency remains uncertain. The present study demonstrated that intravenous iron therapy is associated with reduced fatigue scores; however, the effects on physical function remain poorly defined due to low‐quality evidence. Overall, there is a need for more RCTs at a low risk of bias, which are powered to measure clinically important differences in physical function. Despite affirming empirical evidence, intravenous iron therapy remains a common clinical practice in this demographic, giving additional impetus to future research efforts.

## Conflicts of interest

Lachlan F. Miles is the coordinating principal investigator on a currently running prospective study that has received funds from Vifor Pharma as part of a matched funding arrangement with the Victorian Government. Toby Richards has received grants, personal fees and non‐financial support from Pharmocosmos and Vifor Pharma. He has also received speaker's honoraria from Medtronic. Professor Richards is also a regular speaker at national and international conferences on anaemia, blood transfusion, wound healing and vascular diseases for which he has received expenses for travel, accommodation and sundries. He has worked with several agencies promoting meetings or healthcare, is a director of The Iron Clinic Ltd and director of Veincare London Ltd and is also the Vascular Lead for 18‐week Wait Ltd. Both Katerina Cabolis and Cory Dugan have no conflicts of interest to declare.

## Supporting information


**Appendix S1.** Search strategy
