# Supplementary material for: Systematic review and meta‐analysis of intravenous iron therapy for adults with non‐anaemic iron deficiency: An abridged Cochrane review
Source: J Cachexia Sarcopenia Muscle. 2022 Nov 2;13(6):2637–49. doi: 10.1002/jcsm.13114 (PMC9745472; doi:10.1002/jcsm.13114)
Supplement: Supplementary file 1 — Appendix S1. Search strategy [file JCSM-13-2637-s001.docx]

**Appendices**

**Appendix 1. CENTRAL search strategy**

#1 MeSH descriptor: [Iron] this term only

#2 MeSH descriptor: [Iron Compounds] this term only

#3 MeSH descriptor: [Ferric Compounds] this term only

#4 MeSH descriptor: [Ferrous Compounds] this term only

#5 (iron or ferric* or ferrous)

#6 #1 or #2 or #3 or #4 or #5

#7 MeSH descriptor: [Injections, Intravenous] this term only

#8 (intravenous* or IV or inject*)

#9 #7 or #8

#10 #6 and #9

#11 (nonanemi* or nonanaemi* "non anemi*" or "non anaemi*" or NAID or IDNA)

#12 ("no anemia" or "no anaemia" or "not anemic" or "not anaemic" or "without anemia" or "without anemic" or "without anaemia" or

"without anaemic")

#13 MeSH descriptor: [Iron] this term only and with qualifier(s): [Deficiency - DF]

#14 (iron depletion or iron deficien*)

#15 MeSH descriptor: [Anemia, Iron-Deficiency] this term only and with qualifier(s): [Prevention & control - PC]

#16 #11 or #12 or #13 or #14 or #15

#17#10 and #16

#18 MeSH descriptor: [Infant] explode all trees

#19 MeSH descriptor: [Child] explode all trees

#20 neonat* or newborn* or infant* or child* or schoolchild*

#21 MeSH descriptor: [Pregnancy] explode all trees

#22 pregnan* or postpartum

#23 #18 or #19 or #20 or #21 or #22

#24 #17 not #23

**Appendix 2. MEDLINE Ovid search strategy**

1. Iron/

2. Iron compounds/ or Ferric Compounds/ or Ferrous Compounds/

3. (iron or ferric* or ferrous).ti,ab,kw,rn.

4. or/1-3

5. Injections, Intravenous/

6. (intravenous* or IV or inject*).tw.

7. or/5-6

8. 4 and 7

9. (nonan?emi* or non an?emi* or NAID or IDNA).ab,ti.

10. ("no anemia" or "no anaemia" or "not anemic" or "not anaemic" or "without anemia" or "without anemic" or "without anaemia" or

"without anaemic").ti,ab.

11. Iron/df

12. (iron depletion or iron deficien*).ti,ab,kf.

13. Anemia, Iron-Deficiency/pc

14. or/9-13

15. 8 and 14

16. randomi?ed.ab,ti.

17. randomized controlled trial.pt.

18. controlled clinical trial.pt.

19. placebo.ab.

20. clinical trials as topic.sh.

21. randomly.ab.

22. trial.ti.

23. 16 or 17 or 18 or 19 or 20 or 21 or 22

24. (animals not (humans and animals)).sh.

25. 23 not 24

26. 15 and 25

27. exp infant/

28. exp child/

29. (neonat*or newborn* or infant* or child* or schoolchild*).tw.

30. exp pregnancy/

31. (pregnan* or postpartum).ti.

32. or/28-31

33. 26 not 32

**Appendix 3. Embase Ovid search strategy**

1. iron therapy/

2. iron derivative/

3. ferric ion/

4. ferrous ion/

5. (iron or ferric* or ferrous).ti,ab.

6. or/1-5

7. exp intravenous drug administration/

8. (intravenous* or IV or inject*).tw.

9. or/7-8

10. 6 and 9

11. iron deficiency anemia/

12. iron deficiency/pc [Prevention]

13. (nonan?emi* or non an?emi* or NAID or IDNA).ab,ti.

14. ("no anemia" or "no anaemia" or "not anemic" or "not anaemic" or "without anemia" or "without anemic" or "without anaemia" or

"without anaemic").ti,ab.

15. (iron depletion or iron deficien*).ti,ab.

16. or/11-15

17. 10 and 16

18. exp Randomized Controlled Trial/

19. exp controlled clinical trial/

20. exp controlled study/

21. comparative study/

22. randomi?ed.ab,ti.

23. placebo.ab.

24. *Clinical Trial/

25. exp major clinical study/

26. randomly.ab.

27. (trial or study).ti.

28. 18 or 19 or 20 or 22 or 23 or 24 or 25 or 26 or 27

29. exp animal/ not (exp human/ and exp animal/)

30. 28 not 29

31. 17 and 30

32. exp infant/

33. exp child/

34. (neonat*or newborn* or infant* or child* or schoolchild*).tw.

35. exp pregnancy/

36. exp postpartum hemorrhage/

37. (pregnan* or postpartum).ti.

38. or/32-37

39. 31 not 38

**Appendix 4. Web of Science search strategy**

#16 #14 Not #15

#15 TI= (mouse OR mice OR rat OR rats)

#14 #12 NOT #13

#13 TS=(pregnan* OR postpartum OR neonat* OR newborn* OR infant* OR child* OR schoolchild*)

#12 #11 AND #10

#11 TS=HUMAN

#10 #9 AND #8

#9 TS=((clinical OR control* OR placebo OR random OR randomised OR randomized OR randomly OR random order OR random sequence

OR random allocation OR randomly allocated OR at random) SAME (trial* or group* or study or studies or placebo or controlled))

#8 #5 AND #6 AND #7

#7 TS= (intravenous* OR IV OR inject*)

#6 TS=(ferrous OR ferric OR iron)

#5 #1 OR #2 OR #3 OR #4

#4 TS= ("no anemia" OR "no anaemia" OR "not anemic" OR "not anaemic" OR "without anemia" OR "without anemic" OR "without

anaemia" OR "without anaemic")

#3 TS=(non-anemic OR non-anaemic)

#2 TS="iron depletion"

#1 TS=("iron deficiencies" OR "iron deficiency" OR "iron deficient")

**Appendix 5. ClinicalTrials.gov search strategy**

Condition or disease = (non anaemic OR non anemic OR non anaemia OR non anemia OR no anemia OR no anaemia OR not anemic OR

not anaemic OR without anemia OR without anemic OR without anaemia OR without anaemic) AND Other terms = iron AND (intravenous

OR intravenous OR IV OR injection)

**Appendix 6. WHO ICTRP search strategy**

(non anaemic OR non anemic OR non anaemia OR non anemia OR no anemia OR no anaemia OR not anemic OR not anaemic OR without

anemia OR without anemic OR without anaemia OR without anaemic) = condition AND iron = intervention
